# Diffusion Tensor Imaging Technology to Quantitatively Assess Abnormal Changes in Patients With Thyroid-Associated Ophthalmopathy

**DOI:** 10.3389/fnhum.2021.805945

**Published:** 2022-02-04

**Authors:** Li Rui, Li Jing, Wang Zhenchang

**Affiliations:** Department of Radiology, Beijing Friendship Hospital, Capital Medical University, Beijing, China

**Keywords:** thyroid-associated ophthalmopathy, extraocular muscle, lacrimal gland, diffusion tensor imaging (DTI), MRI

## Abstract

**Objective:**

We aim to investigate the feasibility of using diffusion tensor imaging (DTI) to evaluate changes in extraocular muscles (EOMs) and lacrimal gland (LG) in patients with thyroid-associated ophthalmopathy (TAO) and to evaluate disease severity.

**Materials and Methods:**

A total of 74 participants, including 17 healthy controls (HCs), 22 patients with mild TAO, and 35 patients with moderate-severe TAO, underwent 3-Tesla DTI to measure fractional anisotropy (FA) and mean diffusivity (MD) of the EOMs and LG. Ophthalmological examinations, including visual acuity, exophthalmos, intraocular pressure, and fundoscopy, were performed. FA and MD values were compared among patients with different disease severity. Multiple linear regression was adopted to predict the impact of clinical variables on DTI parameters of orbital soft tissue.

**Results:**

TAO patients’ EOMs and LG showed significantly lower FA values and higher MD compared to HCs’ (*P* < 0.05). Moderate-severe TAO patients’ EOMs and LG had dramatically lower FA and higher MD compared with HCs (*P* < 0.05). In addition, only the DTI parameters of the medial rectus were considerably different between mild and moderate-severe TAO patients (*P* = 0.017, *P* = 0.021). Multiple linear regression showed that disease severity had a significant impact on the DTI parameters of orbital soft tissue.

**Conclusion:**

DTI is a useful tool for detecting microstructural changes in TAO patients’ orbital soft tissue. DTI findings, especially medial rectus DTI parameters, can help to indicate the disease severity in TAO patients.

## Introduction

Thyroid-associated ophthalmopathy (TAO) is an autoimmune inflammatory disease that causes inflammation and fibrosis of orbital fat, extraocular muscles (EOMs) and the lacrimal gland (LG) (Weiler, 2017). The medial rectus and inferior rectus are the most common lesion sites. The expansion of both EOMs and orbital fat is responsible for various clinical symptoms, such as diplopia, proptosis, and restricted eye movement (Drui et al., 2018). A previous study found that approximately 30–45% of TAO patients only showed significant dry eye symptoms, and about 65–75% of TAO patients had dry eye symptoms (Kashkouli et al., 2018). Besides the changes of widened palpebral fissure and increased exophthalmos that accelerate the evaporation of tears, decreased secretion of tears due to LG involvement has been considered as another vital factor (Inoue et al., 2020). TAO is divided into two phases: The active phase of inflammation usually lasts for 6–18 months. It is characterized by monocyte infiltration, edema and fibroblast proliferation. The inactive phase is characterized by orbital soft tissue fibrosis, collagen and fat deposition (Dolman, 2018).

Currently, imaging examination is an important supplement to ophthalmic examination and laboratory examination (Chen L. et al., 2020; Ma et al., 2021). It plays an important role in the clinical diagnosis, evaluation, disease treatment and monitoring TAO patients. Computed tomography (CT) is usually utilized to provide data for an initial diagnosis or pre-surgery evaluation. Magnetic resonance imaging (MRI) has superior soft tissue resolution, T2-weighted imaging (T2WI) signal intensity of the EOMs and dynamic MRI enhancement can assist in diagnosing and staging TAO patients (Politi et al., 2014; Wu et al., 2017). Quantitative measurement of the signal intensity and volume of orbital soft tissue can indicate the response of moderate-severe TAO patients’ to hormone therapy (Hu et al., 2020). Diffusion weighted imaging (DWI) has shown promise in the evaluation of EOMs and LG. Studies have demonstrated a higher ADC in TAO patients’ EOMs and LG compared with HCs (Abdel Razek et al., 2017; Hiwatashi et al., 2018; Razek et al., 2019). In addition, the ADC of EOMs combined with CAS is helpful in guiding clinical decisions (Feeney et al., 2020).

Diffusion tensor imaging (DTI) is an extension of DWI, it can provide quantitative information about the microstructural integrity of highly oriented tissues (Assaf et al., 2019). Fractional anisotropy (FA) and mean diffusivity (MD), as common parameters of DTI, can, respectively, reflect the magnitude and directionality of water diffusion (Norris et al., 2020). DTI has also been widely applied in patients with optic neuritis, glaucoma, and other eye diseases (Kuchling et al., 2017; Colbert et al., 2021). At present, relatively few studies have performed DTI on TAO patients to assess orbital soft tissue microstructural changes (Han et al., 2016; Chen H.H. et al., 2020; Chen et al., 2021). To our knowledge, several studies included EOMs and lacrimal glands in the same research, but only focusing on each separately instead of studying the orbital soft tissues as a whole.

Therefore, the purpose of this study was to evaluate the utility of DTI in detecting microstructural changes in orbit soft tissue in TAO patients and to assess disease severity.

## Materials and Methods

This study was approved by our Institutional Review Board and informed consent was obtained from all subjects.

### Patient Population

From September 2018 to January 2020, 74 subjects were collected, including 57 diagnosed TAO patients and 17 sex- and age-matched HCs. Their diagnosis was according to American Ophthalmological Association standards. Patients who had experienced or had one of the following treatments, including orbital decompression, eyelid surgery, radiotherapy, strabismus surgery, and inadequate image quality were excluded.

The clinical features included age, gender, clinical activity score (CAS) and European Group on Graves’ Orbitopathy (EUGOGO) classification. The CAS is used to evaluate the patient’s disease activity, so as to determine the patient’s sensitivity to hormone therapy (Du et al., 2021). The CAS includes 7 points, one point for each item; a patient with a CAS ≥ 3 is considered in the active phase, and a patient with a CAS < 3 is considered in the inactive phase. 114 eyes (57 TAO patients) were collected in this study, including 56 eyes in the active phase and 58 eyes in the static phase. Disease severity was assessed by adopting 2016 EUGOGO (Perros et al., 2017). The patients were divided into two categories: mild (22 subjects, with 13 females and 9 males), and moderate-severe (35 subjects, with 20 females and 15 males).

### Magnetic Resonance Examination

MRI was performed on a 3.0 Tesla MR scanner (Prisma, Siemens Medical systems, Erlangen, Germany) with a 64-channel head coil. Every volunteer was instructed to look at a fixed point with both eyes closed to reduce motion-related errors. The sagittal 3D thin-layer T1-weighted imaging (T1WI) parameters were as follows: repetition time (TR) 2,530 ms, echo time (TE) 2.98 ms, matrix 250 × 250, 1 mm slice thickness, field of view (FOV), 256 mm × 256 mm. DTI was acquired on an axial plane using the RESOLVE sequence. The scan location line was set parallel to the optic nerve intraorbital segment, and scan parameters were as follows: *b* = 0 and l,000 s/mm^2^; number of diffusion gradient directions (NDGDs), 64; readout segments, 5; TR/TE, 5,500 ms/63 ms; Field of view (FOV)224 mm × 224 mm; matrix 200 × 200;slice thickness 2 mm; the resolution of voxel spacing, 2 mm × 2 mm × 2 mm. The total imaging time was 10 min and 56 s.

Conventional imaging protocols using fast spin echo (FSE) included axial, coronal, and sagittal T2WI imaging with fat suppression, and axial T1WI.

### Imaging Processing

**FIGURE 1:**
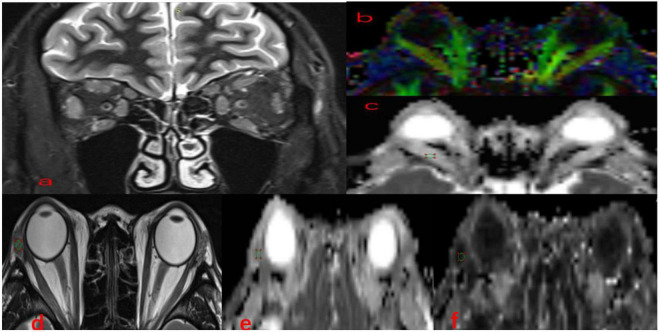
Methods for DTI parameters measurements in EOMs and LG. Coronal **(a)** fat-suppressed T2-weighted images and color-coded FA **(b)**, MD **(c)**, in a 36-year-old man with TAO. A circular region of interest was manually set on each EOM locating the clearest cross-section. Axial **(d)** T2-weighted images, FA **(e)**, MD **(f)** maps of the same patient. A circular region of interest was manually set on each LG indicating the clearest cross-section. TAO, thyroid-associated ophthalmopathy; EOMs, extraocular muscle; DTI, diffusion tensor imaging; LG, lacrimal gland; FA, fractional anisotropy; MD, mean diffusivity.

DTI images were postprocessed using Siemens Prisma 3.0T with Syngo.Via post-processing software. Image analysis was performed in a double-blind manner by two neuroimaging diagnostic physicians who had over 5 years’ working experience in the relevant field, each analysis was performed twice, and the mean values of the two analyses were recorded. The FA, apparent diffusion coefficient (ADC), and TRACEW images were opened in the MR Basic program, and TRACEW images were adjusted to display the clearest level of the EOMs; then, a circular region of interest (ROI) was drawn on EOMs, and the diameter of the ROI was set to 6 mm. After this process, the software could automatically calculate the FA values and the MD values. The study followed a similar method to measure LG. Circular ROIs on the LGs were carefully positioned (Figure 1).

### Statistical Analysis

All numeric data in our research were reported as the mean ± standard deviation, Kolmogorov-Smirnov test was used for analyzing the normality. Since CAS does not conform to the normal distribution, they were reported as median with interquartile range (IQR). One-way analysis of variance (ANOVA) was used to compare age-related differences, the chi-squared test was applied to compare differences regarding medication categories and sex between groups. The Fisher’s exact test was used to compare differences in smoking history between groups. It is worth mentioning that all subjects had the same severity of disease in the right and left eyes, and the average values for left/right eye parameters were calculated. The independent -samples *t*-test was used to compare DTI parameters of EOMs and LG between TAO patients and HCs. Comparisons about the EOMs and LG DTI parameters of participants in the HC, mild, and moderate-severe groups were performed using one-way ANOVA, and intergroup comparisons regarding DTI parameters of orbital soft tissue were conducted using least significant difference (LSD). A correlation matrix was applied to evaluate correlations between EOMs’ and LGs’ DTI parameters. Multi linear regression was performed to assess whether the influencing factors of clinical features (age, gender, disease severity) would affect orbital soft tissue DTI parameters. The odds ratios and their 95% confidence intervals (CIs) were calculated.

Interobserver agreements of quantitative parameters were assessed by the intraclass correlation coefficients (ICCs). Two-way ICCs with random rater assumptions were applied. The results were interpreted as follows: < 0.40, poor; 0.40–0.60, moderate; 0.61–0.80, good; and ≥ 0.81, excellent. A value of *p* < 0.05 was considered statistically significant. All statistical analyses were carried out using the SPSS 20.0 software package.

## Results

The clinical and demographic information are shown in Table 1. No significant differences in age, gender, or smoking among participants in the HCs, mild and moderate-severe groups (*P* > 0.05). In addition, 12 mild patients and 20 moderate -severe patients took antithyroid drugs. There was no statistically significant difference in the orbital soft tissue DTI parameters or clinical features of the left and right eyes of the participants (*p*-values are given in Table 2). Therefore, statistical analysis was performed after calculating the average value of each parameter.

**TABLE 1:** Demographics of the control and TAO groups.

	Normal (*n* = 17)	Mild (*n* = 22)	Moderate to sever (*n* = 35)	F/X^2^	*P*-value
Age	35.4 ± 12.3	39.1 ± 12.3	47.1 ± 14.5	–	0.143^a^
Gender (F/M)	10/7	13/9	20/15	0.026	0.989^b^
Antithyroid drug use, %(n)	–	54.5% (12)	57.1% (20)	0.037	0.847^b^
Smoking,%(n)	17.6% (3)	13.6% (3)	17.1% (6)	–	1.000^c^

*Numeric data are reported as means ± standard deviations. Statistical significance was indicated by p-values< 0.05.*

*F, female; M, male; TAO, thyroid-associated orbitopathy.*

*^a^One-way ANOVA.*

*^b^Chi-squared test.*

*^c^Fisher’s Exact test.*

**TABLE 2:** Comparison of left and right orbital soft tissue DTI parameters and clinical parameters.

Parameters		Left eye	Right eye	Mean	*T*-value	*P*-value
Lateral rectus	FA	0.463 ± 0.059	0.451 ± 0.053	0.457 ± 0.056	1.652	0.103
	MD(×10–2 mm^2^/s)	0.162 ± 0.014	0.164 ± 0.013	0.163 ± 0.011	–1.234	0.221
Medial rectus	FA	0.461 ± 0.069	0.462 ± 0.065	0.462 ± 0.050	–0.040	0.968
	MD(×10–2 mm^2^/s)	0.159 ± 0.013	0.159 ± 0.013	0.159 ± 0.013	–0.011	0.991
Lacrimal gland	FA	0.383 ± 0.046	0.380 ± 0.048	0.382 ± 0.037	0.400	0690
	MD(×10–2 mm^2^/s)	0.138 ± 0.010	1	0.138 ± 0.008	0.281	0.779
Clinical parameters	Exophthalmometry (mm)	19.7 ± 3.0	19.2 ± 2.8	19.5 ± 2.9	0.416	0.680
	CAS(range)	3.8(1–6)	3.5(1–6)	3.7	1.756	0.088
	Intraocular pressure (mmHg)	18.2 ± 3.9	17.9 ± 3.4	18.1 ± 3.7	1.318	0.196

*Numeric data are reported as means ± standard deviations; CAS are reported as median with interquartile range (IQR).*

*FA, fractional anisotropy; MD, mean diffusivity; CAS, clinical activity score.*

Excellent inter-observer reproducibility were obtained when measuring all DTI parameters (ICC ranged from 0.898 to 0.995). The FA of EOMs and LG in patients with TAO was significantly lower, the MD was dramatically higher than that of the HCs (FA: P_Lateral rectus_ = 0.038, P_Medial rectus_ = 0.009, P_Lacrimal gland_ = 0.027; MD: P_Lateral rectus_ = 0.046, P_Medial rectus_ = 0.002, P_Lacrimal gland_ = 0.001) (Table 3).

**TABLE 3:** DTI parameters of orbital soft tissue between TAO and HC.

Parameters		HC	TAO	*P*
Lateral rectus	FA	0.487 ± 0.066	0.450 ± 0.035	0.038
	MD(× 10–2 mm^2^/s)	0.157 ± 0.015	0.165 ± 0.009	0.046
Medial rectus	FA	0.490 ± 0.058	0.454 ± 0.046	0.009
	MD(× 10–2 mm^2^/s)	0.153 ± 0.009	0.161 ± 0.009	0.002
Lacrimal gland	FA	0.399 ± 0.049	0.376 ± 0.031	0.027
	MD(× 10–2 mm^2^/s)	0.134 ± 0.004	0.139 ± 0.008	0.001

*Numeric data are reported as means ± standard deviations.*

*HC, healthy control group; TAO, thyroid-associated ophthalmopathy; FA, fractional anisotropy; MD, mean diffusivity.*

**FIGURE 2:**
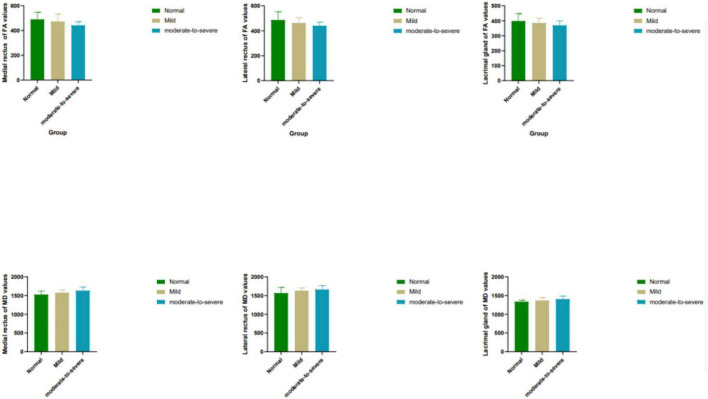
Box plots show comparisons of all DTI parameters in EOMs and LG between groups. Units of MD are × 10^– 6^ mm^2^/s. FA, fractional anisotropy; MD, mean diffusivity.

Moderate- severe TAO patients showed significantly lower FA and higher MD compared with HCs’ and those from the mild group (all *P* < 0.05). In addition, comparing the moderate-severe patients with mild patients, the former’s medial rectus’ FA were considerably lower and its MD were significantly higher (*P* = 0.017, *P* = 0.021) (Figure 2 and Table 4).

**TABLE 4:** DTI parameters of orbital soft tissue and clinical parameters in the normal, mild and moderate-to-severe TAO groups.

Parameters		Normal	Mild	Moderate-to-severe	*F*-value	*P*-value	*P*-value		
							a	b	c
Lateral rectus	FA	0.487 ± 0.066	0.463 ± 0.042	0.441 ± 0.028	6.569	0.002	0.094	0.001	0.066
	MD (× 10–2 mm^2^/s)	0.157 ± 0.015	0.163 ± 0.008	0.166 ± 0.010	4.293	0.017	0.075	0.005	0.301
Medial rectus	FA	0.490 ± 0.058	0.473 ± 0.060	0.442 ± 0.029	6.775	0.002	0.273	0.001	0.017
	MD (×10–2 mm^2^/s)	0.154 ± 0.017	0.156 ± 0.008	0.163 ± 0.010	4.998	0.009	0.558	0.006	0.021
Lacrimal gland	FA	0.400 ± 0.049	0.386 ± 0.031	0.370 ± 0.030	3.911	0.024	0.264	0.008	0.112
	MD (×10–2 mm^2^/s)	0.134 ± 0.004	0.137 ± 0.008	0.141 ± 0.008	5.523	0.006	0.159	0.002	0.071
Clinical parameters	CAS	0 (0, 0)	2.00 (1.00, 2.00)	4.00 (3.00, 5.00)	51.884	<0.001	0.001	<0.001	0.002
	Exophthalmometry(mm)	13.1 ± 0.8	18.2 ± 2.9	19.7 ± 3.0	35.927	<0.001	<0.001	<0.001	0.051
	Intraocular pressure (mmHg)	14.6 ± 2.7	17.9 ± 3.1	18.2 ± 4.0	6.687	0.002	0.005	0.001	0.701

*Numeric data are reported as means ± standard deviations. Continuous data are reported as median (interquartile range). Statistical significance was indicated by p-values < 0.05.*

*HC, healthy control group; TAO, thyroid-associated ophthalmopathy; FA, fractional anisotropy; MD, mean diffusivity.*

*a, normal compared to mild; b, norma compared to moderate-to-severe; c, Mild compared to moderate to severe.*

*Bonferroni correction P< 0.05/3 = 0.017.*

The correlation matrix showed that there was a positive correlation between the DTI parameters of the medial rectus and that of the lateral rectus (P_FA_ = 0.002, P_MD_ = 0.042). No correlation was discovered between EOMs and LG DTI parameters. Detailed p and r values are presented in Table 5. Multi linear regression demonstrated that disease severity was a significant influencing factor for orbital soft tissue DTI parameters (P_FA_< 0.01, P_MD_ = 0.050), whereas gender was the influencing factor which affect orbital soft tissue MD only (*P* = 0.017) (Table 6).

**TABLE 5:** Correlations matrix of orbital soft tissue DTI parameters.

		DTI-FA			DTI-MD		
		Medial rectus	Lateral rectus	Lacrimal gland	Medial rectus	Lateral rectus	Lacrimal gland
DTI-FA	Medial rectus		*r* = 0.360 *P* = 0.002	*r* = 0.005 *P* = 0.967			
	Lateral rectus			*r* = −0.215 *P* = 0.066			
	Lacrimal gland						
DTI-MD	Medial rectus					*r* = 0.237 *P* = 0.042	*r* = 0.129 *P* = 0.274
	Lateral rectus						*r* = −0.188 *P* = 0.109
	Lacrimal gland						

*FA, fractional anisotropy; MD, mean diffusivity.*

**TABLE 6:** Multivariate logistic regression analysis results for predicting impact to orbital soft tissue DTI parameters.

	Parameters	β coefficient	SE	Odds ratio(95% CI)	*p*-value
Orbital soft tissue FA values	Disease severity	–0.637	10.315	1.708(−86.812 to −45.667)	<0.001
	Gender	–0.122	15.924	1.022(−52.461 to 11.058)	0.198
	Age	0.123	0.614	2.134(−0.466 to 1.983)	0.221
Orbital soft tissue MD values	Disease severity	–0.236	29.629	2.023(−118.256 to −0.069)	0.050
	Gender	–0.275	45.742	2.200(−203.268 to −20.808)	0.017
	Age	0.032	1.763	1.606(−3.043 to 3.991)	0.789

*SE, standard error; CI, confidence interval; FA, fractional anisotropy; MD, mean diffusivity.*

## Discussion

In this study, comparing TAO patients with the HC ones, the FA of the EOMs and LG were significantly lower, while the MD were significantly higher. This result was in agreement with the findings of previous studies (Chen H.H. et al., 2020; Chen et al., 2021). Moreover, comparing mild TAO patients with moderate-severe patients, the medial rectus’ FA were dramatically lower while its MD were higher. Previous studies (Abdel Razek et al., 2017) have found that the medial rectus was more affected than the lateral rectus muscle; therefore, our research suggests that DTI can well detect microstructural changes in the medial rectus of TAO patients.

DTI is a non-invasive MRI technology which is very sensitive to water molecules’ micromovement. Thus, it can provide quantitative information of water molecule diffusion movement (Jeon et al., 2018). Common parameters of DTI include FA and MD values. The MD can reflect the magnitude of water diffusion dominated by interstitial space. Researchers found an inverse correlation between tumor cells and MD values (Razek et al., 2018; Abdel Razek et al., 2019). FA can represent the directionality of water molecules, hence it can reflect tissue’s integrity of microstructural architectures (Gholizadeh et al., 2019). The FA value is a number between 0 and 1. It is equal to 0 under isotropic conditions, and it approaches 1 in case of extreme directional inequality.

TAO patients’ orbital soft tissues were thought to experience two phase: the initial acute inflammation is characterized by hyaluronic acid accumulation and mononuclear inflammatory cell infiltration. Subsequently, chronic inactive phase was featured with interstitial fibrosis, and fatty infiltration (Taylor et al., 2020). In our study, most TAO patients were in the active phase. During this phase, the mononuclear cell infiltration, fibroblast proliferation and edema in EOMs and LG causes an increase in the extracellular space, which also enhances the ability of water molecules to diffuse. Since MD reflects the magnitude of water diffusion, an escalating trend of MD values can be acquired. The acute inflammation stage is also associated with cell lysis and fibrous disruption (Men et al., 2021). FA can reflect integrity of microstructural architectures, as mentioned. In our research, EOMs and LG of TAO patients exhibited lower FA compared with those in HCs.

In our study, the correlations matrix of orbital soft tissue DTI parameters revealed that the DTI parameters of the EOMs had a positive correlation, however, there was no correlation between the LG and the EOMs. This result indicates that in the process of mononuclear cell infiltration, fibroblast proliferation, and edema in EOMs, the medial rectus and the lateral rectus affect each other. The interaction between the extraocular muscles and the lacrimal gland in the development of the disease is not obvious. Several previous researches (Lee et al., 2018; Razek et al., 2019; Chen H.H. et al., 2020; Chen et al., 2021) have examined extraocular muscles and lacrimal gland DTI separately, but these studies did not discuss the relevance of orbital soft tissue in the development of the disease. In addition, as previous studies state (Cao et al., 2021; Neag and Smith, 2021), TAO mainly involves orbital fat, extraocular muscles, and lacrimal gland. In this study, we placed the lacrimal gland and extraocular muscles in the same picture, that is the orbital soft tissues, and we calculated their DTI parameters. Multivariate analysis along with clinical factors revealed that disease severity significantly impacted orbital soft tissue DTI parameters. We believe it is a unique finding which has not yet been discovered.

Several previous studies have focused on the use of functional MRI technique, such as measuring EOMs or LG apparent diffusion coefficient (ADC) values, to diagnose TAO (Abdel Razek et al., 2017; Feeney et al., 2020; Chen et al., 2021). Other studies have mainly focused on assessing disease activity and predicting the response to glucocorticoid therapy using methods such as calculating the T1 mapping or T2 mapping values of EOMs (Das et al., 2019; Chen L. et al., 2020; Hu et al., 2021). Few reports have focused on functional MRI of orbital soft tissue in patients with different severities of TAO. To our knowledge, accurately staging for patients with TAO is crucial for clinical decision making, especially for moderate-severe TAO patients (Farag et al., 2021). In this research, DTI parameters of the medial rectus are sensitive imaging indicators, which could distinguish mild from moderate-severe TAO patients.

Our study had several limitations. First, the study cohort of this study was relatively small, so expanding the sample size in future studies is necessary. Second, No common standard has been settled for DTI acquisition technology and for post-processing. This may lead to poor compatibility among different research results. In addition, since the scan for DTI sequence is axial, the lack of information on inferior EOMs might be a major drawback of our study. We will continue to optimize the DTI sequence in future studies. Furthermore, the patients were not followed up after their treatment in this study, and these patients will also be included in the future, so we can have a more comprehensive understanding of TAO.

## Conclusion

In conclusion, our results suggest that DTI technology can well detect the changes in orbital soft tissue microstructure. It can also provide more objective imaging biological indicators for clinical applications. DTI parameters of the medial rectus, especially the FA of medial EOMs, can serve as an effective indicator of disease severity.

## Data Availability Statement

The original contributions presented in the study are included in the article/supplementary material, further inquiries can be directed to the corresponding author/s.

## Ethics Statement

The studies involving human participants were reviewed and approved by the Department of Ethics Committee, Beijing Friendship Hospital, Capital Medical University. The patients/participants provided their written informed consent to participate in this study.

## Author Contributions

LR, WZ, and LJ: conception and design. WZ and LJ: administrative support. LR: provision of study materials or patients, collection, and assembly of data. LR and LJ: data analysis and interpretation. All authors: manuscript writing, final approval of manuscript.

## Conflict of Interest

The authors declare that the research was conducted in the absence of any commercial or financial relationships that could be construed as a potential conflict of interest.

## Publisher’s Note

All claims expressed in this article are solely those of the authors and do not necessarily represent those of their affiliated organizations, or those of the publisher, the editors and the reviewers. Any product that may be evaluated in this article, or claim that may be made by its manufacturer, is not guaranteed or endorsed by the publisher.
